# Evaluation of the ablation margin of hepatocellular carcinoma using CEUS-CT/MR image fusion in a phantom model and in patients

**DOI:** 10.1186/s12885-017-3061-7

**Published:** 2017-01-19

**Authors:** Kai Li, Zhongzhen Su, Erjiao Xu, Qiannan Huang, Qingjing Zeng, Rongqin Zheng

**Affiliations:** Department of Ultrasound, The Third Affiliated Hospital of Sun Yat-sen University, Guangzhou, 510630 Guangdong Province People’s Republic of China

**Keywords:** Tumor ablation, Phantom model, CEUS, CT, Image fusion

## Abstract

**Background:**

To assess the accuracy of contrast-enhanced ultrasound (CEUS)-CT/MR image fusion in evaluating the radiofrequency ablative margin (AM) of hepatocellular carcinoma (HCC) based on a custom-made phantom model and in HCC patients.

**Methods:**

Twenty-four phantoms were randomly divided into a complete ablation group (*n* = 6) and an incomplete ablation group (*n* = 18). After radiofrequency ablation (RFA), the AM was evaluated using ultrasound (US)-CT image fusion, and the results were compared with the AM results that were directly measured in a gross specimen. CEUS-CT/MR image fusion and CT-CT / MR-MR image fusion were used to evaluate the AM in 37 tumors from 33 HCC patients who underwent RFA.

**Results:**

The sensitivity, specificity, and accuracy of US-CT image fusion for evaluating AM in the phantom model were 93.8, 85.7 and 91.3%, respectively. The maximal thicknesses of the residual AM were 3.5 ± 2.0 mm and 3.2 ± 2.0 mm in the US-CT image fusion and gross specimen, respectively. No significant difference was observed between the US-CT image fusion and direct measurements of the AM of HCC. In the clinical study, the success rate of the AM evaluation was 100% for both CEUS-CT/MR and CT-CT/MR-MR, and the duration was 8.5 ± 2.8 min (range: 4–12 min) and 13.5 ± 4.5 min (range: 8–16 min) for CEUS-CT/MR and CT-CT/MR-MR, respectively. The sensitivity, specificity, and accuracy of CEUS-CT/MR imaging for evaluating the AM were 100.0, 80.0, and 90.0%, respectively.

**Conclusions:**

A phantom model composed of carrageenan gel and additives was suitable for the evaluation of HCC AM. CEUS-CT/MR image fusion can be used to evaluate HCC AM with high accuracy.

## Background

Radiofrequency ablation (RFA) is a radical treatment for hepatocellular carcinoma (HCC) and has a relatively low risk [1, 2]. However, recent studies have shown that HCC patients undergoing RFA have a higher rate of local tumor progression (LTP) compared with HCC patients treated with resection [3–6]. Independent factors associated with LTP include tumor size, sub-capsular location, blood vessel proximity, and an insufficient ablation margin (AM) [7–15]. The term “ablative margin” refers to the 0.5 to 1.0-cm-wide region of normal tissue around the tumor that should ideally be removed during tumor ablation [16, 17]. Therefore, AM is one of the most important factors for the prediction of LTP in HCC patients after RFA [18–20]. However, regular medical imaging methods, including CT, MR, and contrast-enhanced ultrasound (CEUS), are not able to accurately evaluate AM because the tumor and surrounding normal liver tissue mix and merge in the ablative area, and the boundary between normal tissue and the ablative area is difficult to identify. Thus, using the current imaging methods, it is challenging to determine whether the zone of ablation encompasses the range of the AM around the index tumor.

In recent years, novel medical imaging methods have been explored to assess AM in HCC patients after ablation, including CT-CT image fusion [21, 22], MR-MR image fusion [23, 24], contrast-enhanced ultrasound (CEUS)-CT/MR image fusion [18, 25], MR with impaired clearance of ferucarbotran [26, 27], and MR with gadolinium ethoxybenzyl diethylene triamine pentaacetic acid [28]. Our group has reported that CEUS-CT/MR image fusion, which can be applied intraoperatively, is useful for assessing AM in HCC patients receiving ablation [25]. An accurate evaluation of AM based on CEUS-CT/MR image fusion allows physicians to perform supplementary ablation, increasing the number of adequate AM and reducing the probability of LTP. However, direct measurement of AM in HCC patients is not feasible because the gross specimen is usually unavailable in ablation patients. Therefore, the rate of LTP has been widely used as the standard to evaluate the accuracy of AM in most studies. Tumor tissues that are not covered by AM during HCC ablation are usually a major cause of LTP. However, AM is not the only independent factor associated with LTP after ablation. Therefore, the accuracy of CEUS-CT/MR image fusion in assessing AM should be further evaluated.

In this study, we established a phantom model to evaluate AM based on US-CT image fusion. The aim of this study was to assess the accuracy of CEUS-CT/MR image fusion for the evaluation of the AM of liver tumors, both in an in vitro phantom model and in the clinic. The gross specimen of the phantom after RFA was used as a gold standard. The clinical AM results obtained using CEUS-CT/MR image fusion and MR-MR/CT-CT image fusion were compared.

## Methods

### Materials

The materials used to construct the phantom model included carrageenan (Dehui Marine Biological Technology Cor., Ltd., Qingdao, China) and a number of additives such as oral ultrasonic contrast agent (Hangzhou Huqingyutang Medical Technology Cor., Ltd., Hangzhou, China), gastric window contrast agent (Huqingyutang Cor., Hangzhou, China), milk, and Congo red.

### Establishment of the phantom model

**Fig. 1:**
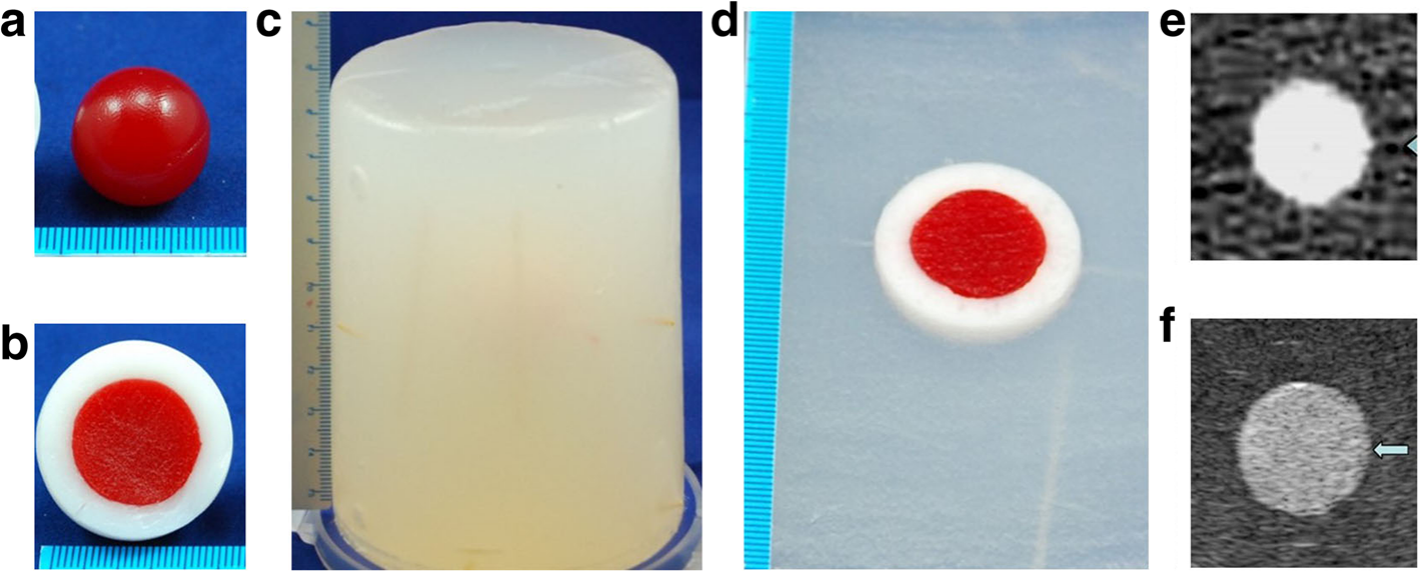
**a** A spherical tumor model (2 cm in diameter) made of carrageenan in red. **b** Section of the AM model: the carrageenan tumor model surrounded by the 5-mm AM gel in white. **c** Cylindrical-shaped parenchyma model (10 cm in height and 10 cm in diameter of the upper and lower plane). **d** Section of the cylindrical-shaped parenchyma model. **e** CT image showing the tumor. The scope of AM could not accurately evaluated. **f** US image showing the tumor. The boundary between the tumor and AM gel was not clear

The phantom model included a spherical tumor model (2 cm in diameter, Fig. 1a), an AM model (a 5-mm layer of AM gel around the tumor model, Fig. 1b), and a cylindrical parenchyma model (10 cm in height and diameter, Fig. 1c), in which the AM model was embedded (Fig. 1d). Bamboo sticks in the parenchyma model were used as registration and positional marks.

### Model testing

The shape, height, and gradient of the phantom model were tested at 1 h, 6 h, and 12 h after construction of the model. The structure of the phantom model and the position of the marks were checked using both ultrasound and observation of the gross specimen.

### Study grouping

A total of 24 phantom models were randomly divided into two groups, including a complete ablation group (*n* = 6) in which the ablative area was covered by AM and an incomplete ablation group (*n* = 18) in which the ablative area was not covered by AM.

### Radiofrequency ablation

RFA was performed with a cooled-tip RFA system (Covidien, Mansfield, MA, USA) using a 17-gauge, internally cooled-tip electrode with a 3-cm tip. A MyLab Twice ultrasound machine (Esoate, Genoa, Italy) and a linear probe LA332 (frequency range from 3 to 11 MHz) with imaging fusion (Virtual Navigation System) and three-dimensional software were employed for ultrasound guidance and exploration.

An ablative area model was established by ablating the tumor model. Electrodes were inserted into the cylindrical model parenchyma via ultrasound guidance by an experienced ultrasound interventional doctor. The RFA was set in impedance mode with maximum output. According to our pilot studies, the duration of ablation was 4 and 6 min for the incomplete and complete ablation groups, respectively. After ablation, the liver tumor model together with the AM model and a portion of the parenchyma model were melted and mixed. An ablative area model was established after cooling and solidifying the melted gel.

### Evaluation of AM by US-CT image fusion

**Fig. 2:**
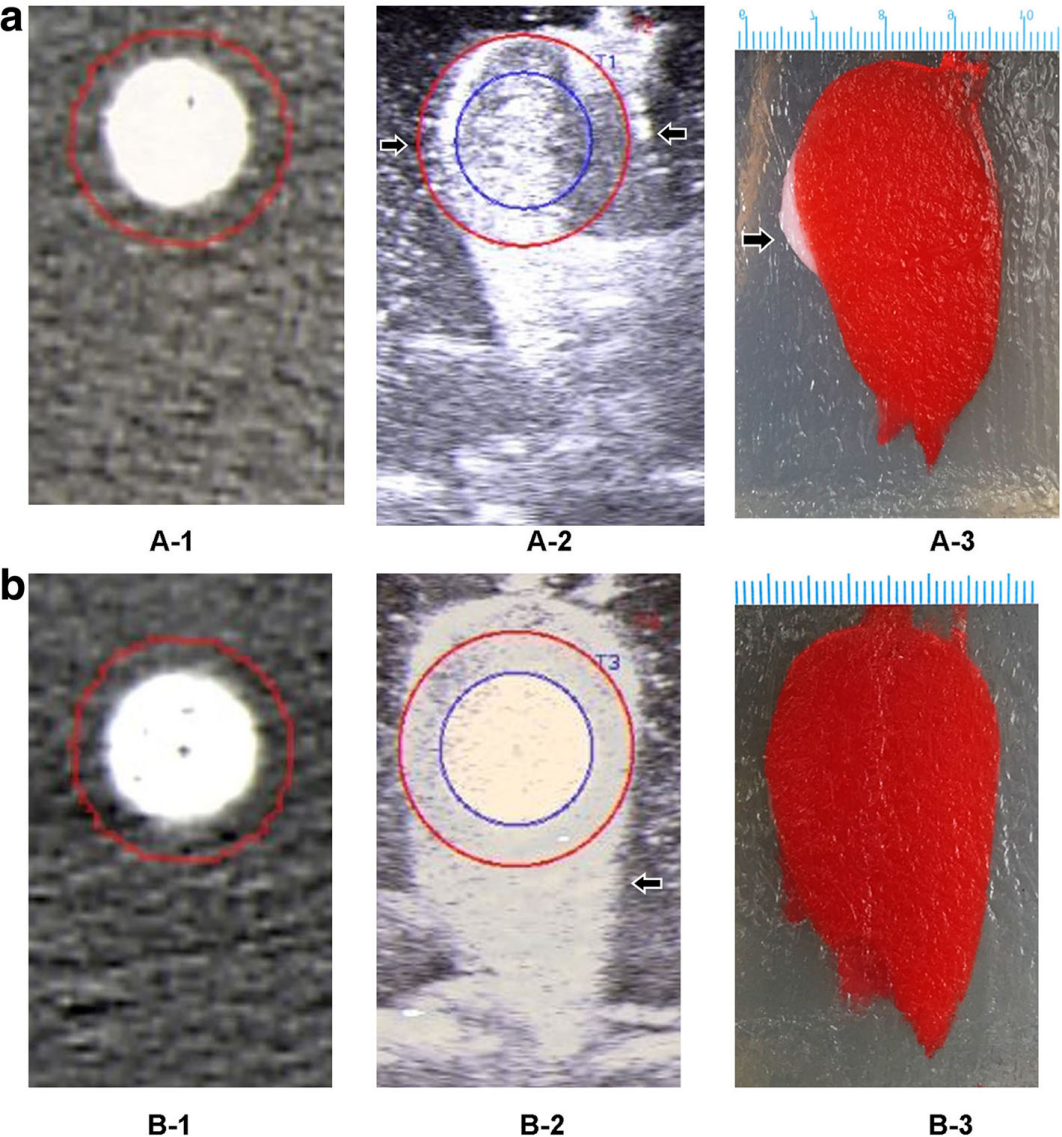
(A-1) and (B-1): CT images of the models. (A-2) and (B-2): US-CT image fusion of the area of ablation (*left arrow*). The AM was not fully encompassed by the area of ablation (*right arrow*, A-2). The tumor and AM were completely encompassed by the area of ablation (B-2). (A-3): The AM was not completely encompassed by the area of ablation in the gross specimen (*left arrow*). (B-3): The AM was completely encompassed by the area of ablation in the gross specimen. The tumor is shown in blue, and the AM gel is indicated in red (5 mm)

Each phantom CT scan was performed without contrast medium prior to ablation. The CT scan was performed using a 64-row multi-detector CT scanner (VCT 64 slices; GE Medical Systems). The following CT parameters were applied to acquire dynamic data: 1-s gantry rotation time, 120 kV, 80 mA, acquisition in 264 transverse mode (64 sections per gantry rotation), and 2.5-mm reconstructed section thickness (Fig. 2A-1, B-1). The model was positioned horizontally with the guidance of a horizontal laser instrument.

Image fusion was performed by an experienced ultrasound doctor who was blinded to the ablation. A series of CT data in DICOM format were uploaded in fusion mode into the ultrasound system to automatically generate images. The areas of the tumor model and 5-mm AM in the 3D CT images were outlined using red and blue circles, respectively. At the beginning of image fusion, the registration marks in the phantom models were used to choose one transverse section of the CT image and the ultrasound image of the same section. The CT and ultrasound images were then overlapped and fused. After registration of this section, additional fine tuning was performed to enable a more precise adaptation. The distance between the CT and ultrasound images of the same registration mark in overlapping mode could be measured and used as the error of image fusion. A successful image fusion was defined when the error of image fusion of all registration marks was less than 2 mm. Otherwise, the registration was repeated. The image fusion was considered a failure if a successful fusion could not be achieved after three attempts. In overlapping mode, inclusion of the tumor within the ablative area of the model and the AM mode could be decided. The position and thickness of the thickest part of the AM model was recorded if the AM was not completely covered (Fig. 2A-2, B-2).

### Evaluation of the AM in the gross specimen

**Fig. 3:**
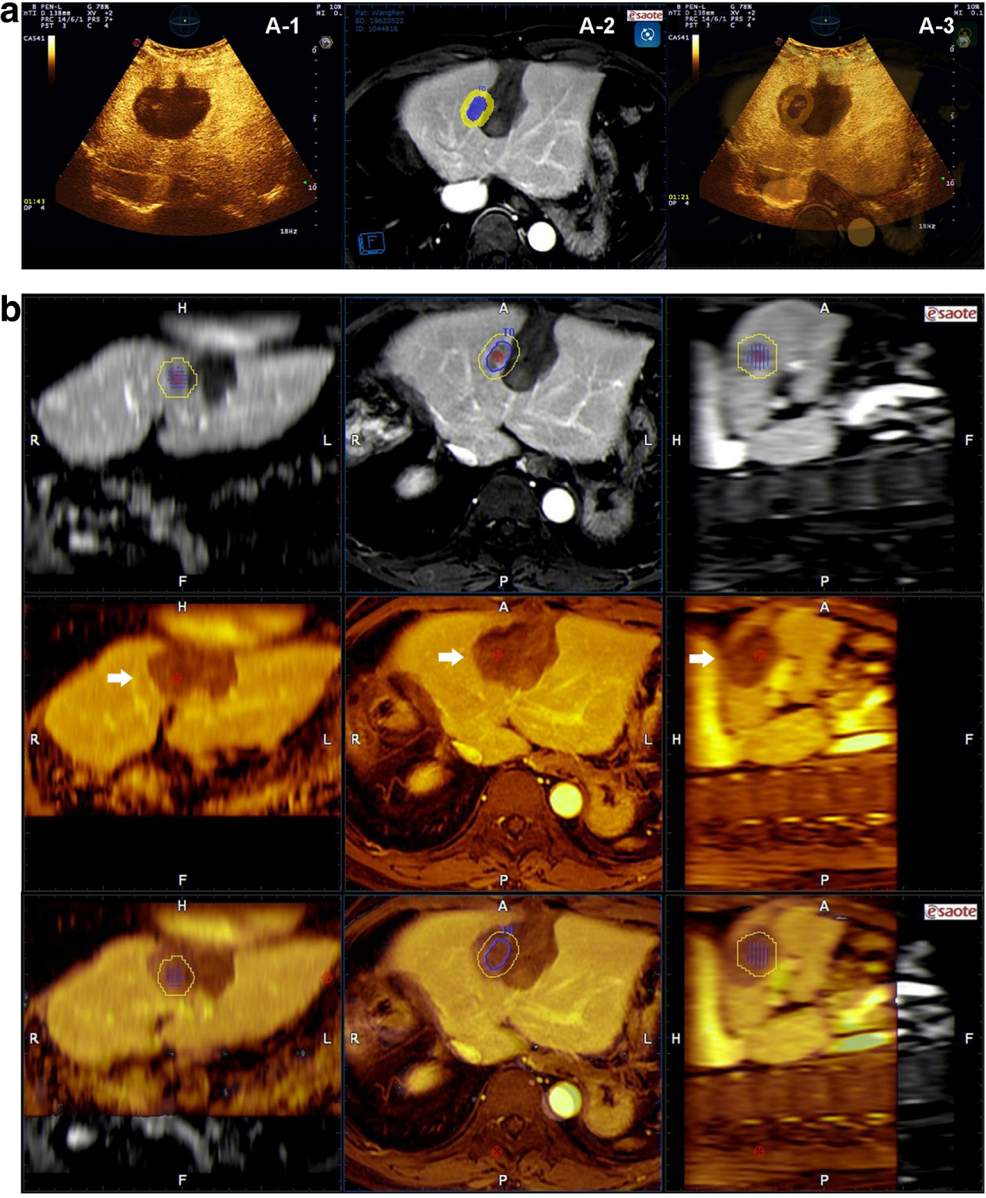
Medical images of case 1 in the clinical study. (A-1) CUES image of the area of ablation (dark). (A-2). Preoperative MR image (tumor shown in blue, and 5-mm AM shown in yellow). (A-3) CEUS-MR fusion image. The tumor and 5-mm AM were fully encompassed by the area of ablation. **b** MR-MR fusion image of case 1. *Upper panel*: preoperative MR images (tumor shown in blue, and 5-mm AM shown in yellow). *Middle panel*: postoperative MR images (arrow, ablation area shown in dark color). *Lower panel*: MR-MR fusion images showing that the tumor and the 5-mm AM were fully encompassed by the area of ablation

The phantom was cut along the section showing the thickest residue in the AM model by image fusion. Whether the AM model had been fully ablated was examined, and the maximal thickness of the AM model residue was measured. In addition, the results of the US-CT image fusion and gross specimen were compared to calculate the sensitivity, specificity, and accuracy of US-CT image fusion for the evaluation of AM model residue (Fig. 2A-3, B-3).

### Ethics statement and study populations

This study was approved by the Institute Research Medical Ethics Committee of the Third Affiliated Hospital of Sun Yat-Sen University and was in compliance with the Declaration of Helsinki. Informed consent was obtained from all participants. From January 2014 to April 2014, a total of 33 HCC patients who underwent RFA in our hospital were enrolled in this study. All liver lesions meeting the Milan criteria were pathologically or clinically diagnosed as HCC [29]. Inclusion criteria were as follows: the ablation zone of the tumors was evaluated by CEUS-CT/MR image fusion after RFA. Exclusion criteria were as follows: 1) failure to obtain CT/MR data in DICOM format from the patient preoperatively; 2) the patient did not receive a CT/MR examination 1–2 months after RFA; 3) different image methods (CT and MR) were applied preoperatively and postoperatively, precluding image fusion; 4) ultrasound and CT/MR images could not be successfully fused; 5) the patient was allergic to ultrasound contrast agents.

### RFA

We used the same cooled-tip RFA system in the phantom model research for HCC patient RFA. The ablation was performed under endotracheal anesthesia. All RFA procedures were performed by two experienced ultrasound physicians with more than 5 years of RFA experience. According to the routine examination, previously determined plan, and multiple needle ablations for larger tumors, all HCC lesions, including the 5-mm AM, were successfully ablated. CEUS-CT/MR image fusion was performed approximately 10 min after RFA to evaluate the efficacy of RFA and to guide the supplementary ablation.

### CEUS-CT/MR image fusion

CEUS-CT/MR image fusion was performed using the MyLab Twice (Esaote, Italy) ultrasound unit and convex array transducer CA431 (4–10 MHz) 10–15 min after ablation. Virtual Navigator was the image fusion program and CnTI (MI <0.05) was the imaging technique for contrast-enhanced ultrasound in the ultrasound unit. SonoVue (Bracco, Italy) was used as the contrast agent. For each application, 2.4 ml of SonoVue was administered through the antecubital vein and flushed by 5 ml of normal saline.

**Fig. 4:**
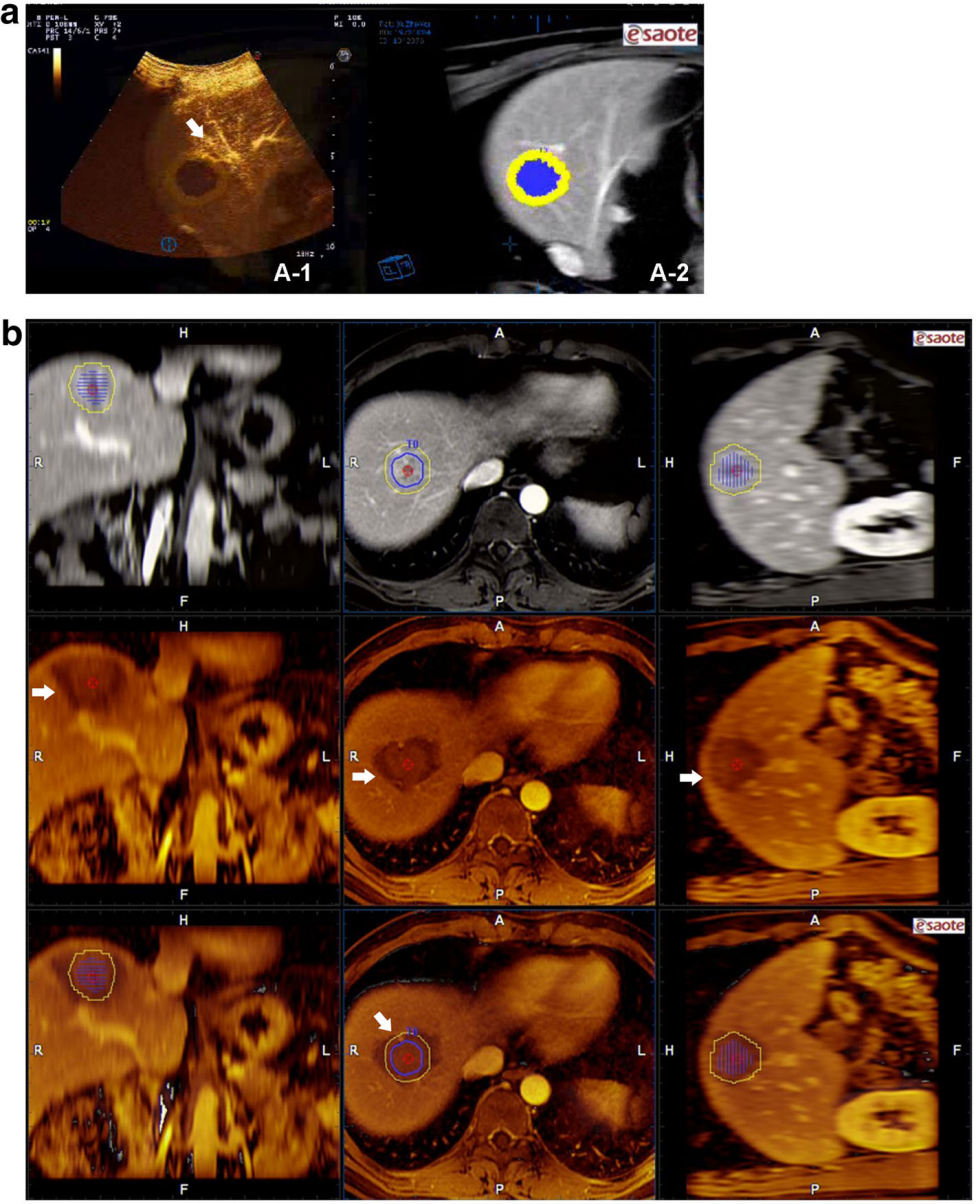
Medical images of case 2 in the clinical study. (A-2) Preoperative MR image (tumor shown in blue and 5-mm AM in yellow). (A-1) CEUS-MR fusion image showing that the area of ablation encompassed the tumor but not the entire AM due to the influence of vessels (*white arrow*). **b** MR-MR fusion image from the same patient. *Upper panel*: preoperative MR images (tumor shown in blue and 5-mm AM in yellow). *Middle panel*: postoperative MR images (arrow, area of ablation shown in dark color). *Lower panel*: MR-MR fusion images showing that the tumor and AM were not fully encompassed by the area of ablation (white arrow)

The method of CEUS- CT/MR image fusion used in this study had been reported in our former article [18]. The CT/MR image series in DICOM format were transferred into the navigation system and 3D image volume was generated. Different colors were used to outline the tumor and 5-mm AM (Figs. 3A-2, 4A-2). After planar registration, more precise fusion was acquired through additional refinement. Then CEUS was performed and the image of CEUS was overlapped with the CT/MR image to see whether the area of CEUS had covered the tumor as well as the AM region.

### CT-CT/MR-MR image fusion

Contrast-enhanced CT/MR was performed 1 month before and after the RFA for all patients. AM was further evaluated using *CT-CT/*MR-MR image fusion if the CT/MR at 1 month after RFA revealed that the lesion was completely ablated.

One CT/MR portal or delayed phase series with a clearly demonstrated hepatic vessel and ablative area before RFA in DICOM format was transferred into the navigation system in MyLab Twice. One month after RFA, another series of CT/MR images were also imported into the image fusion system. The system then automatically displayed six pictures in two rows: the upper row included the transaction, coronal and vertical section CT/MR images before RFA, and the lower row showed the corresponding CT/MR images after RFA. The HCC lesion in the CT/MR before RFA was manually outlined, and then a 5-mm AM was set automatically in different colors (Figs. 3b, 4b).

Image registration was performed by aligning two overlaid CT/MR images. Translation and rotation were performed in three reformed planes to maximize the image similarity around the HCC lesion and the area of ablation. The hepatic vein, hepatic artery portal complex and hepatic contour near the lesion were used as landmarks for fine adjustments to obtain a satisfactory registration. The pre- and post-RFA CT/MR images were then overlapped to assess whether the ablative area encompassed the HCC lesion and the 5-mm AM. The standards of complete registration included complete matching of three corresponding anatomic landmarks adjacent to the tumor, and the offset was less than 5 mm in each plane. Failed registration was determined when the above standards were not achieved after three attempts. The time spent on registration for each lesion and the success rate of MR-MR image fusion were recorded. The results of the CT-CT/MR-MR image fusion as standard were used to evaluate the accuracy of the CEUS-CT/MR image fusion.

### Data analysis

The analyzed data included 1) the duration required for the US-CT image fusion (phantom model study), CEUS-CT/MR image fusion, and MR-MR image fusion (clinical study); 2) the success rate of US-CT image fusion (phantom model study), CEUS-CT/MR image fusion and CT-CT/MR-MR image fusion (clinical study); 3) the accuracy rate of the assessment, including the coincidence rates of the assessment of complete ablation between US-CT image fusion and the gross specimen (phantom model study), and between CEUS-CT/MR image fusion and CT-CT/MR-MR image fusion; and 4) the maximum thickness of the residual AM in the US-CT image fusion and the gross specimen.

### Statistical analyses

Statistical analyses were performed using SPSS for Microsoft Windows (version 13.0; SPSS Inc. Chicago, IL, USA). The data are the mean ± standard deviation (range). The paired *t* test was used to compare the maximum thickness of the residual AM in the US-CT image fusion and the gross specimen. A *P* value less than 0.05 was considered significant.

## Results

### Successful establishment of the phantom models

The echogenicity, density, and color of different components of the phantom models met the requirements (Table 1). The appearance, height, and gradient of the phantom models were stable at 1 h, 6 h, and 12 h after model production.

**Table 1:** The ultrasound echo, CT density and color of the phantom models

Model	Ultrasound echo	CT density	Color
Parenchyma model	Hypoechoic^a^	Low density^b^	Colorless semitransparent
Tumor model	Hyperechoic	High density	Red opaque
AM model	Hypoechoic^a^	Low density^b^	Milky opaque
Ablative area model	Slightly hyperechoic	--------	Carnation semitransparent

^a^These two had the same echogenecity, ^b^these two had the same density

### US-CT image fusion detected residual AM with high sensitivity, specificity, and accuracy in the phantom models

Of the 24 phantom models, one phantom was accidentally damaged, and 23 phantoms were used in all follow-up experiments. Image fusion was successfully obtained from the 23 phantoms. The success rate of image fusion was 100% (23/23). The average time used for image fusion was 5–12 min (median = 7 min). Compared with the gross specimen, the sensitivity, specificity, and accuracy of the US-CT image fusion for the detection of residual AM were 100.0, 93.8 and 95.7%, respectively (Table 2). In one case, the US-CT image fusion showed that AM was completely encompassed by the ablative area, but a 1-mm residual AM was still observed in the gross specimen. The maximal thicknesses of residual AM calculated by the US-CT image fusion and measured in the gross specimen were 3.5 ± 2.0 mm and 3.2 ± 2.0 mm, respectively, which suggested that there was no significant difference (*P* = 0.705).

**Table 2:** The results of AM evaluated by US-CT image fusion and gross specimen (*P* > 0.05)

		Gross specimen		Total
		AM covered	AM not covered	
US-CT image fusion	AM covered	7	1	8
	AM not covered	0	15	15
	Total	7	16	23

### CEUS-CT/MR image fusion revealed residual AM with a high sensitivity, specificity, and accuracy in the clinical study

A total of 30 tumors from 26 patients were enrolled in the clinical study. The clinical characteristics of the participants and HCC lesions are shown in Table 3. Seven patients were excluded from the study, including three patients without a postoperative CT/MR examination and three patients with inconsistent preoperative and postoperative imaging methods. In addition, one patient was excluded from the clinical study due to the formation of a local abscess in the ablation zone after RFA, which could bias the AM assessment.

**Table 3:** The clinical characteristics of the patients and HCC lesions

Characteristics	Number
Gender (Male/Female)	26/0
Age (mean ± standard deviation, years)	
Virus hepatitis/alcoholic liver disease/no diffuse hepatic disease	26/0/0
Liver cirrhosis (yes/no)	/
Tumor number (1/>1)	22/4
Tumor diameter (mean ± SD, mm)	19.1 ± 6.5

*HCC* hepatocellular carcinoma, *M* median, *QR* interquartile range

CT-CT image fusion was conducted for one lesion, and MR-MR image fusion was applied for the remaining lesions. The success rate of CEUS-CT/MR image fusion and CT-CT/MR-MR image fusion were both 100% (30/30). The duration was 8.5 ± 2.8 min (range: 4–12 min) and 13.5 ± 4.5 min (range: 8–16 min) for the CEUS-CT/MR and CT-CT/MR-MR image fusions, respectively. The results of the AM evaluation based on CEUS-CT/MR and MR/MR image fusions are shown in Table 4. An inadequate AM was caused by blood vessels in seven cases (46.7%) and an inadequate ablation zone in eight cases (53.3%). Compared with CT-CT/MR-MR image fusion, the sensitivity, specificity, and accuracy of CEUS-CT/MR image fusion for the evaluation of AM were 100.0, 80.0, and 90.0%, respectively.

**Table 4:** The results of AM evaluated by CEUS-CT/MR image fusion and MR/MR image fusion

		MR/MR image fusion		Total
		AM covered	AM not covered	
CEUS-CT/MR image fusion	AM covered	15	3	18
	AM not covered	0	12	12
	Total	15	15	30

## Discussion

Phantoms have been widely used to evaluate the effects of thermal treatments. Previous studies, however, have mainly focused on other topics, such as temperature monitoring, energy distribution, the relationship between RF and electrical conductivity, and development of the heating algorithm applied in drug delivery. It is not clear whether phantoms are good models for the evaluation of AM using a CEUS-CT/MR image fusion system. Therefore, in the present study, we established a phantom model to evaluate AM using CEUS-CT/MR image fusion. In our study, we found that the peculiarly thermal invertibility and thermal sensitivity of the carrageenan gel were useful for distinguishing the ablative zone, the remaining ‘tumor’, and the AM after RFA. While the carrageenan hybrid gel used in the present study was not the best material for the evaluation of thermal ablation, especially for temperature variation and energy distribution, we took full advantage of the physical properties of the carrageenan gel. To the best of our knowledge, this is the first report to assess the accuracy of the evaluation of complete RF using an image fusion system that matched pre-RFA and post-RFA images in a tissue-mimicking phantom.

We developed a hybrid gel phantom using carrageenan and other substances, which have a number of important properties, i.e., sufficient strength, low fragility, and low cost. Carrageenan, a high-molecular-weight polysaccharide extracted from red algae, consists of repeating galactose and 3,6-anhydrogalactose units linked by alternating α-1,3- and β-1,4-glycosidic linkages. Carrageenan can be used in a phantom model because it is inexpensive and safe, as well as broadly applied for the production of gel products and other foods. Additive agents played significant roles in the construction, imaging, and observation by the naked eye. For example, NaCl was added to the carrageenan gels to adjust the gel conductivity. US contrast agent and iodipin were used to improve the echo or to enhance attenuation. In addition, Congo red, an indicator used for the diagnosis of amyloidosis by generating a bright and distinct red color, was easily distinguished from the opaque gel. The red color may have infiltrated the peripheral gel due to the diffusion of Congo red. However, we believe that Congo red has no influence on the results of the AM evaluation if the whole procedure, including manufacturing, RFA, and assessment of the ablative zone, is completed within 6 h. Using carrageenan together with other substances, we were able to create a large and robust phantom model with excellent shape retention. The turbidity and low fragility of the carrageenan gel in the phantom model ensured accurate image registration. In addition, we designed a phantom that mimicked the tumor lesion (i.e., a visible sphere) to assess the AM using the fusion imaging system after RF. The easy heating and coagulation of the phantom model allowed us to assess the post-RFA destructive zone more accurately. Improved visualization of the target by US and CT, as well as the distinct color of the materials, also improved the ablation assessment. Therefore, the phantom model established herein was successfully used for the evaluation of the AM of the HCC tumor.

The present experimental study results suggest that the US-CT fusion image system can be used to accurately and effectively evaluate AM. However, in one case, US-CT image fusion revealed that the AM was completely covered by the ablative area, and even less than a 1-mm AM was observed in the gross specimen. The false-positive case could be caused by registration error and magnetic positioning system error. Given that the phantom model was idealized to evaluate AM, the feasibility and accuracy was further validated in a clinical assessment.

In our clinical study, the sensitivity, specificity, and accuracy of CEUS-CT/MR image fusion for the evaluation of AM were 100.0, 80.0 and 90.0%, respectively, suggesting that CEUS-CT/MR image fusion is a good tool for evaluating AM after HCC ablation. CEUS-CT/MR image fusion combines the advantages of CEUS and CT/MR and expands the use of both imaging methods, including the high spatial contrast resolution of CT/MR and real-time guidance, accessibility, and practicality of ultrasound. In addition, CEUS-CT/MR image fusion greatly improves intraoperative AM evaluation and the localization of tumor lesions compared with MR-MR image fusion.

We discovered three false-positive cases in the clinical study, which might be due to the ability of CEUS to only demonstrate blood perfusion of tissues rather than necrosis. The high temperature of the local zone of ablation may cause swollen tissues and small vessel occlusion, limiting the infiltration of blood into the ablated area. However, occluded small vessels can be reperfused after the local tissue temperature decreases, suggesting that the ablation zone may be over-measured by intraoperative CEUS.

The present study has several limitations. First, the phantom model cannot completely mimic dynamic tissues and organs, such as respiratory movements, which may reduce the accuracy of the registration for image fusion and affect the imaging assessment. While the assessment was performed successfully in the idealized phantom model, some unknown problems may be present in the in vivo experiments, which must be identified and resolved. Second, the phantom models applied in the present study were used for US-CT image fusion, whereas most of the clinical cases were evaluated by CEUS-CT/MR image fusion. Thus, the accuracy of the results may be biased. Third, all of the patients enrolled in this study were male, which may also bias the results. Therefore, further studies with more experience, a larger sampling size and better technology are needed.

## Conclusions

In conclusion, we successfully established a phantom model for the evaluation of AM using US-CT/MR image fusion. Our results suggest that US-CT/MR image fusion is an accurate approach for evaluating AM after tumor ablation based on both an in vitro model and a clinical study.

## Abbreviations

AM: Ablative margin
CEUS: Contrast-enhanced ultrasound
HCC: Hepatocellular carcinoma
LTP: Local tumor progression
RFA: Radiofrequency ablation
